# Depletion of Mitochondrial DNA in Differentiated Retinal Pigment Epithelial Cells

**DOI:** 10.1038/s41598-019-51761-1

**Published:** 2019-10-25

**Authors:** Xinqian Hu, Melissa A. Calton, Shibo Tang, Douglas Vollrath

**Affiliations:** 1 State Key Laboratory of Ophthalmology, Zhongshan Ophthalmic Center, Sun Yat-sen University, Guangzhou, 510060 China; 2 Department of Genetics, Stanford University School of Medicine, Stanford, CA 94305 USA; 3 AIER School of Ophthalmology, Central South University, Changsha, China; 4 AIER Eye Institute, Changsha, China; 5 CAS Center for Excellence in Brain Science and Intelligence Technology, Chinese Academy of Sciences, Shanghai, 200031 China

**Keywords:** Senescence, Retinal diseases, Medical research

## Abstract

We investigated the effects of treating differentiated retinal pigment epithelial (RPE) cells with didanosine (ddI), which is associated with retinopathy in individuals with HIV/AIDS. We hypothesized that such treatment would cause depletion of mitochondrial DNA and provide insight into the consequences of degradation of RPE mitochondrial function in aging and disease. Treatment of differentiated ARPE-19 or human primary RPE cells with 200 µM ddI for 6–24 days was not cytotoxic but caused up to 60% depletion of mitochondrial DNA, and a similar reduction in mitochondrial membrane potential and NDUFA9 protein abundance. Mitochondrial DNA-depleted RPE cells demonstrated enhanced aerobic glycolysis by extracellular flux analysis, increased AMP kinase activation, reduced mTOR activity, and increased resistance to cell death in response to treatment with the oxidant, sodium iodate. We conclude that ddI-mediated mitochondrial DNA depletion promotes a glycolytic shift in differentiated RPE cells and enhances resistance to oxidative damage. Our use of ddI treatment to induce progressive depletion of mitochondrial DNA in differentiated human RPE cells should be widely applicable for other studies aimed at understanding RPE mitochondrial dysfunction in aging and disease.

## Introduction

Mitochondria serve a myriad of important functions in cells. Mitochondrial DNA mutations accumulate with age in the human brain and retina^1,2^, and inherited and acquired mitochondrial DNA mutations are associated with photoreceptor degeneration. Macular retinopathy is a feature of some syndromes caused by inherited mitochondrial mutations^3–6^, and acquired damage to the mitochondrial DNA of long-lived retinal pigment epithelial (RPE) cells has been implicated in the pathogenesis of age-related macular degeneration (AMD)^7–9^.

A number of studies have investigated the consequences of RPE mitochondrial dysfunction by using undifferentiated, proliferating or confluent cultured cell lines^10–12^. The response of cells to loss of mitochondrial DNA can vary depending upon the differentiated functions of individual cell types^10^. Several studies used RPE lines lacking mitochondrial DNA (ρ^0^)^10,11^, generated by repeated passaging in medium containing the mutagen ethidium bromide^13^. Primary RPE cells lose their differentiated characteristics upon repeated passaging and undergo an epithelial to mesenchymal-like transition^14,15^. While repeated passaging is feasible for immortalized RPE lines, ρ^0^ cells have altered intermediate metabolism, as evidenced by their auxotrophy for uridine and a requirement for exogenous pyruvate to synthesize aspartate^13,16^. Moreover, differentiated RPE cultures more faithfully recapitulate the *in vivo* physiology and cell biology of this multi-functional epithelial tissue^17^. We therefore sought to study the consequences of RPE mitochondrial dysfunction using cultured, differentiated cells.

Some nucleotide reverse transcriptase inhibitors (NRTIs) used to treat individuals with acquired immunodeficiency syndrome (AIDS) inhibit polymerase (pol-γ), the enzyme responsible for replication and repair of mitochondrial DNA^18^. Prolonged treatment with such NRTIs results in decreased mitochondrial DNA relative to nuclear DNA in both mice and humans^19–21^. NRTIs inhibit pol-γ to varying degrees. Treatment with one of the most potent inhibitors, didanosine (2′, 3′-dideoxyinosine, ddI)^22,23^, a purine nucleoside analog, has been linked to the development of retinopathy in children and adults suffering from HIV/AIDS^24–28^. Retinal lesions appear as areas of RPE mottling and atrophy, usually in the midperiphery, but macular involvement has also been described^29^. Histological examination of postmortem tissue from an individual with ddI retinopathy implicated the RPE as the nidus of retinal pathology^25^. Oxidative stress is an important cause of retinal degeneration^30^. However, the role of oxidative stress in ddI induced retinopathy is not clear.

Mitochondrial genomes replicate randomly and independently of the cell cycle^31^, even in differentiated tissues and quiescent cultured cells^32,33^. Treatment of differentiated human renal proximal tubule epithelial cells with ddI significantly reduced the relative content of mitochondrial DNA after three weeks^22^. While the specifics of RPE mitochondrial DNA turnover are obscure, we hypothesized that exposure of cultured, non-proliferating RPE cells to ddI would result in loss of mitochondrial DNA. To test this hypothesis, we treated cultured, differentiated human RPE cells with ddI and assessed the effects, with the aim of elucidating the pathogenesis of ddI-induced retinopathy and gaining insight into the consequences of RPE mitochondrial DNA dysfunction in aging and disease.

## Methods

### Cell culture

Immortalized human retinal pigment epithelial cells (ARPE-19) were cultured initially as described^34^. Cells were seeded at a density of 3 × 10^5^ cells/cm^2^ on 12-well transwell inserts (Corning Costar 12 mm insert, 0.4 μm polyester membrane) coated with Matrigel (BD Biosciences). For differentiation, after one week the culture medium was changed to differentiation medium: DMEM/F12 medium with 15 mM HEPES and L-glutamine (Invitrogen), 1% FBS, antibiotic/antimycotic (Invitrogen), 1 ng/mL bFGF (Invitrogen), 10^−8^ M retinoic acid (Sigma-Aldrich), 10 ng/mL hydrocortisone (Sigma-Aldrich), 0.5× of transferrin insulin selenium supplement (Invitrogen) at 37 °C with 10% CO_2_. Cells were cultured in differentiation medium for 4–6 weeks prior to drug treatment, with medium changes 3 times per week. Primary human fetal RPE (hfRPE) cells were isolated according to the methods of Maminishkis and Miller^35^, and plated onto human extracellular matrix-coated Corning 12-well transwell inserts in medium as described with 5% fetal bovine serum^36^. Cells were allowed to differentiate for at least 5 months before beginning experiments.

### ddI treatment

Differentiated ARPE-19 cells were treated in triplicate with ddI (Videx, NDC 0087-6632-41) at doses of 0, 50, 100, and 200 μM, for 6, 12, or 24 days. A 105.8 mM stock of ddI dissolved in sterile phosphate-buffered saline (PBS) was stored at 4 °C, shielded from light. The stock was warmed to 37 °C prior to dilution in culture medium. Medium with ddI was changed three times a week. Differentiated hfRPE cells were cultured with 0 or 200 μM ddI in triplicate for 6 days before DNA was extracted or assays were performed. This concentration was based on a previous study^22^ and was 5 to 20-fold higher than levels used clinically because the clinical symptoms take many years to develop.

### DNA extraction

Total cellular DNA was extracted from cultured cells by a phenol/chloroform/isoamyl alcohol protocol shown to effectively recover mitochondrial DNA^37^, with minor modifications. Washed pellets were resuspended in 100 μL nuclease-free water, and the DNA concentration was estimated by spectrophotometry (NanoDrop, ND-1000).

### Mitochondrial/nuclear DNA ratio

Genomic segments of a mitochondrial-encoded gene (*MT*-*ND1*) and a nuclear-encoded gene (β-actin, *ACTB*) were quantified by PCR. The *MT*-*ND1* primers used were 5′-ATG GCC AAC CTC CTA CTC CT-3′ (forward) and 5′-CTA CAA CGT TGG GGC CTT T-3′ (reverse), and the *ACTB* primers were 5′-ACT CTT CCA GCC TTC CTT CC-3′ (forward) and 5′-GGC AGG ACT TAG CTT CCA CA-3′ (reverse)^38^. PCR was performed using 20 ng of DNA, 250 nM for each primer, and SYBR green master mix with the following protocol: 94 °C for 5 min, followed by 35 cycles through 94 °C × 10 s, 62 °C × 30 s, and 72 °C × 10 s, then 72 °C × 4 min. Each sample was done in triplicate. The relative mitochondrial/nuclear (mt/n) DNA ratio of each sample was calculated.

### Transepithelial resistance

Transepithelial resistance (TER) was measured on cultured hfRPE cells using a two-electrode epithelial voltohmmeter (EVOM; World Precision Instruments). Three measurements were taken and averaged per well.

### Cytotoxicity assay

We used a lactate dehydrogenase (LDH) assay kit (Thermo Scientific Pierce LDH Cytotoxicity Assay Kit) to measure cytotoxicity according to the manufacturer’s instructions.

### Mitochondrial membrane potential (MMP)

We trypsinized and collected differentiated ARPE-19 cells treated or not with ddI 200 μM in triplicate for 24 days, stained them with an MMP sensitive dye, DiOC_2_(3), according to the manufacturer’s instructions (Invitrogen) and analyzed the cells by flow cytometry (BD Accuri, C6). DiOC_2_(3) accumulates in mitochondria with active/high MMPs as red fluorescence (FL3-A) and shifts to green fluorescence (FL1-A) when MMP is decreased. We used the MMP disrupter, CCCP, included in the kit, and unstained cells to set the gates.

### Cellular energetics

We used a Seahorse XFp analyzer (Agilent) to measure oxygen consumption rate (OCR), an indicator of mitochondrial respiration, and extracellular acidification rate (ECAR), an indicator of aerobic glycolysis. Differentiated ARPE-19 cells treated with or without 200 μM ddI for 24 days were trypsinized and reseeded into XFp cell culture miniplates (Agilent) at 40,000 cells per well one day prior to the assay. On the day of an experiment, we switched to XF Assay Medium (Agilent) supplied with 10 mM glucose, 1 mM sodium pyruvate, and 4 mM L-glutamine (Mitochondrial stress test medium), or 4 mM L-glutamine (Glycolysis stress test medium). After 60 minutes of incubation in a non-CO_2_ incubator, we measured OCR and ECAR in different programs (Mitochondrial stress test or Glycolysis stress test). The following compounds were injected for a mitochondrial stress test at final concentrations of: oligomycin (2.0 μM), FCCP (1.5 μM), and rotenone/myxothiazol (2.0 μM). For a glycolysis stress test, we used final concentrations of glucose (10 mM), oligomycin (2.0 μM), and 2-DG (50 mM). There were 3 wells per plate per condition. Three separate measurements were taken after addition of each reagent to the medium. Proteins were collected from each well after the assay and individually quantified using a BCA Protein Assay Kit (Pierce), and OCR and ECAR values were normalized to the total amount of protein for each well. All parameter values were calculated per well according to manufacture instructions. Both Mitochondrial stress test and Glycolysis stress tests were done three times on different days.

### Immunoblot

Protein lysates were prepared as previously described^39^ from differentiated ARPE-19 cells treated with or without 200 μM ddI in triplicate for 24 days. Total protein for each sample was quantified with a BCA kit and an equal amount of protein from each sample was separated by 4–15% gradient SDS-PAGE. Protein transfer and chemiluminescence detection were done as previously described^40^. The primary antibodies used and dilutions were as follows: anti-AMPKα (Cell Signaling Technology, #2603), 1:1000, anti-pAMPKα (Cell Signaling Technology, #2535S), 1:1000, anti-NDUFA9 (Invitrogen, #459100), 1:500, anti-S6 (Cell Signaling Technology), 1:1000, anti-pS6 (Cell Signaling Technology), 1:1000, anti-γ-tubulin (Sigma Aldrich), 1:5000. The secondary antibodies used were HRP-conjugated anti-mouse and anti-rabbit (Jackson ImmunoResearch), 1:10000. Densitometry was performed with ImageJ (NIH).

### Response to oxidative stress

Differentiated ARPE-19 cells were treated with or without ddI 200 μM for 24 days, and then exposed to 15 mM sodium iodate or vehicle for 6 hours. The supernatants were collected and LDH cytotoxicity assays were performed. Cell viability was assessed by the alamarBlue assay (Thermo Fisher) according to the manufacturer’s instructions. Each treatment was done in triplicate.

### Statistics

GraphPad Prism 6.0 was used to assess statistical significance by an unpaired 2-tailed Student’s *t* test, unless otherwise stated. Data in figures represent mean ± SD, unless otherwise noted. *p ≤ 0.05, **p ≤ 0.01, ***p ≤ 0.001, ****p ≤ 0.0001.

## Results

### ddI treatment depletes mitochondrial DNA in differentiated RPE cells

**Figure 1:**
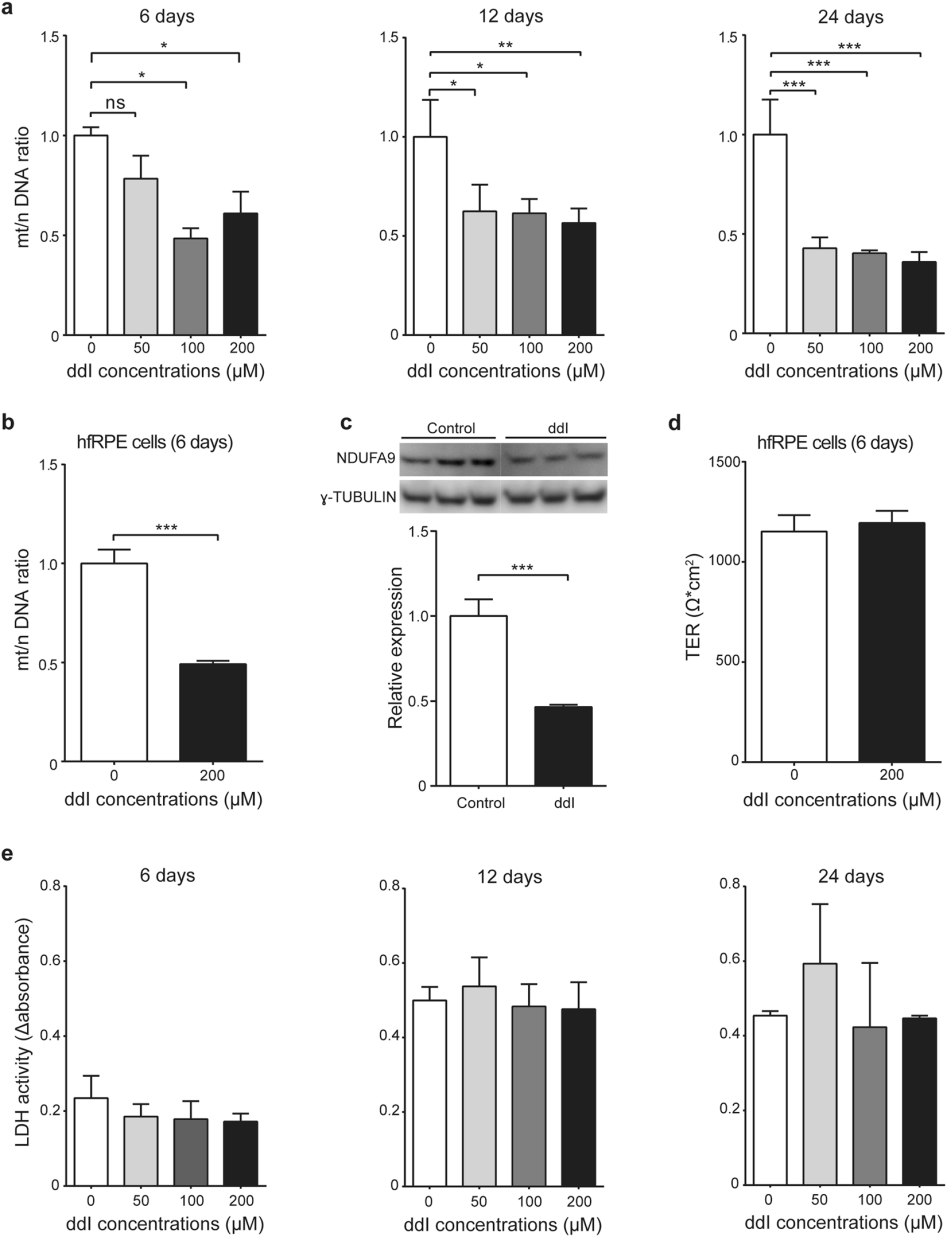
Mitochondrial DNA depletion using ddI. mt/n DNA ratios of (**a**) differentiated ARPE-19 cells treated for various durations (6 days: 0 vs. 50 µM: p = 0.1958; 0 vs. 100 µM: p = 0.0115; 0 vs. 200 µM: p = 0.0311; 12 days: 0 vs. 50 µM: p = 0.0192; 0 vs. 100 µM: p = 0.0167; 0 vs. 200 µM: p = 0.0086; 24 days: 0 vs. 50 µM: p = 0.0002; 0 vs. 100 µM: p = 0.0002; 0 vs. 200 µM: p = 0.0001) and (**b**) hfRPE cells treated for 6 days (p = 0.0003). (**c**) Immunoblot (top) and quantification (bottom) of protein lysates from differentiated ARPE-19 cells treated with 200 μM ddI for 24 days (p = 0.0007). (**d**) average TER measurements from three different hfRPE lines untreated or treated with 200 μM ddI for 24 days; paired Student’s *t* test (p = 0.4874). (**e**) LDH activity of media from differentiated ARPE-19 treated with ddI for various durations (6 days: p = 0.3345; 12 days: p = 0.6616; 24 days: p = 0.3423). n = 3 for each group; (a & e) assessed by one-way ANOVA with Bonferroni’s multiple comparison test.

To assess the effect of ddI on non-proliferating RPE cells, we treated confluent cultures of differentiated cells with various doses of the drug for 6, 12, or 24 days. We quantified the mt/n DNA ratio in both differentiated ARPE-19 and hfRPE cells. ddI treatment results in a progressive reduction in mt/n DNA ratio in ARPE-19 cells (Fig. 1a), to as low as 40% of the control after 24 days with 200 µM, and a similar reduction in hfRPE cells treated for 6 days with the same dose (Fig. 1b). There were no significant differences in the threshold cycle values between treated and untreated samples for *ACTB*, the nuclear gene amplified from these samples (data not shown), suggesting an approximately 60% decrease in the absolute amount of cellular mitochondrial DNA following ddI treatment. Differentiated ARPE-19 cells treated with 200 µM ddI for 24 days exhibit a similar 60% reduction in the amount of NDUFA9 protein, a nuclear-encoded component of electron transport complex I (Fig. 1c, full-length blots in Supplementary Figure 1).

We saw no evidence of cellular toxicity such as loss of adhesion or changes in morphology under low magnification bright field microscopy for any of the samples. The TER of differentiated hfRPE treated for 24 days with 200 µM ddI remained high and indistinguishable from controls (Fig. 1d). Similarly, there were no significant differences in LDH activity in culture media from controls and ddI-treated ARPE-19 cells for various doses and durations (Fig. 1e). Together, these results indicate that ddI causes a significant depletion of mitochondrial DNA and protein in differentiated RPE cells without causing cytotoxicity.

### ddI-treated differentiated RPE cells have impaired mitochondrial functions

#### Lower MMP

**Figure 2:**
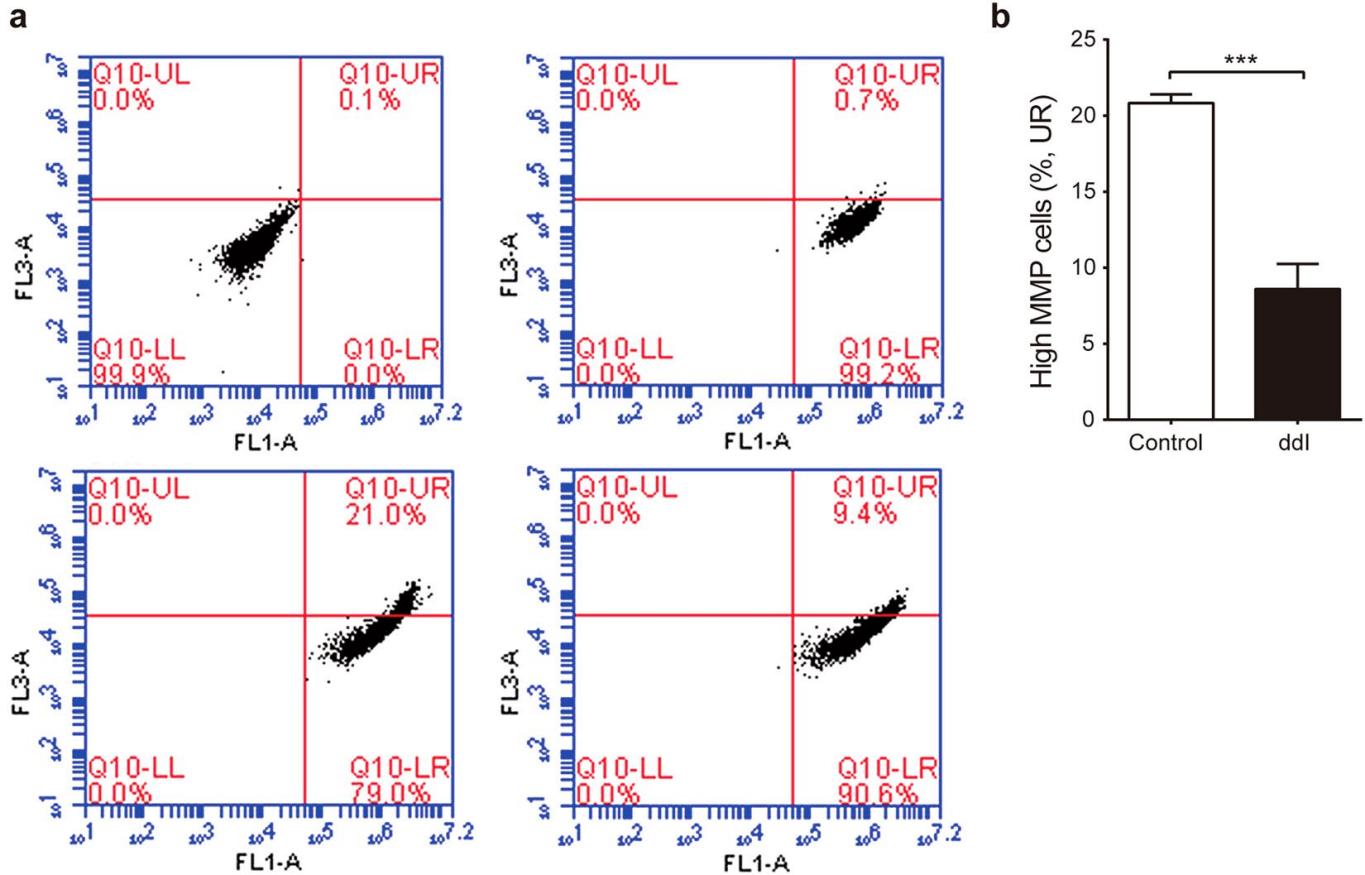
Mitochondrial Membrane Potential (MMP) after ddI treatment. (**a**) Representative flow cytometry of differentiated ARPE-19 stained with DiOC_2_(3): no dye control (upper left panel), uncoupler control CCCP (upper right panel), no ddI (lower left panel), and 200 μM ddI for 24 days (lower right panel). (**b**) Percentage of high MMP for ddI-treated and untreated cells (p = 0.0003). n = 3 for each group.

MMP generated by the asymmetric transport of protons during mitochondrial respiration is an important measure of mitochondrial function, and mitochondrial dysfunction often manifests as decreased MMP. We quantified MMP in differentiated ARPE-19 cells after 24 days of treatment with 200 μM ddI. The fraction of cells with high MMP decreased from about 21% to about 9% in treated cells (Fig. 2a,b), an approximately 60% reduction.

#### Glycolytic shift

**Figure 3:**
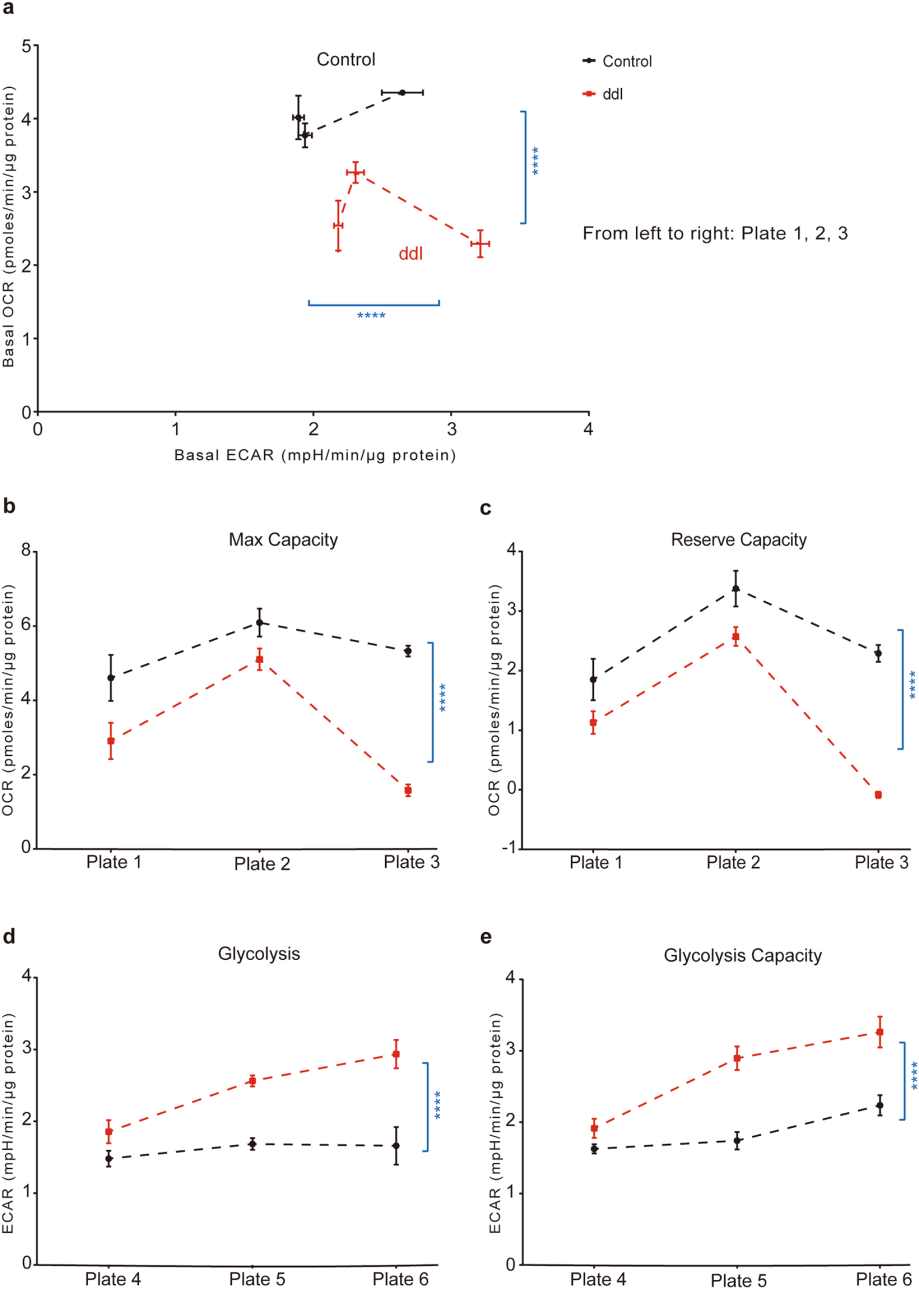
Cellular energetics of differentiated ARPE-19 after 200 μM ddI (24 days) treatment. (**a**–**c**) Mitochondrial stress test; (**d**,**e**) Glycolysis stress test. (**a**) Ratios of basal OCR vs. ECAR in control and treated cells are significantly different (p < 0.0001 for both OCR and ECAR). (**b**) Max capacities and (**c**) Reserve **c**apacities of control and treated cells are significantly different (p < 0.0001 in both (**b**,**c**)). (**d**) Glycolysis and (**e**) Glycolysis capaciti**e**s of control and treated cells are significantly different (p < 0.0001 in both (**d**,**e**)). Black bars: controls, red bars: 200 μM ddI. There were three wells per plate per condition. n = 3 for each group; same experiments were done three times on different days (different plates). Parameters of ddI treated and control groups were analyzed by two-way ANOVA.

A reduction in mitochondrial function might manifest as a change in the balance between the two primary modes of cellular ATP production, oxidative phosphorylation (OXPHOS) and aerobic glycolysis. We found that treatment of differentiated ARPE-19 cells for 24 days with 200 μM ddI decreased OCR and increased ECAR (Fig. 3a) at baseline, indicating a shift toward aerobic glycolysis and lactate secretion. We performed a mitochondrial stress test on the same cells to obtain a more detailed picture of mitochondrial energy function. Cells treated with ddI showed significant reductions in maximum capacity and reserve capacity (Fig. 3b,c). The reduction in mitochondrial respiratory function was accompanied in treated ARPE-19 cells by an increase in specific parameters assessed by a glycolysis stress test^41^, including glycolysis and glycolytic capacity (Fig. 3d,e). These shifts toward increased aerobic glycolysis are consistent with a decrease in the ability of ddI-treated RPE cells to meet energy requirements via OXPHOS.

### AMPK activation and mTORC1 inhibition in ddI-treated ARPE-19 cells

**Figure 4:**
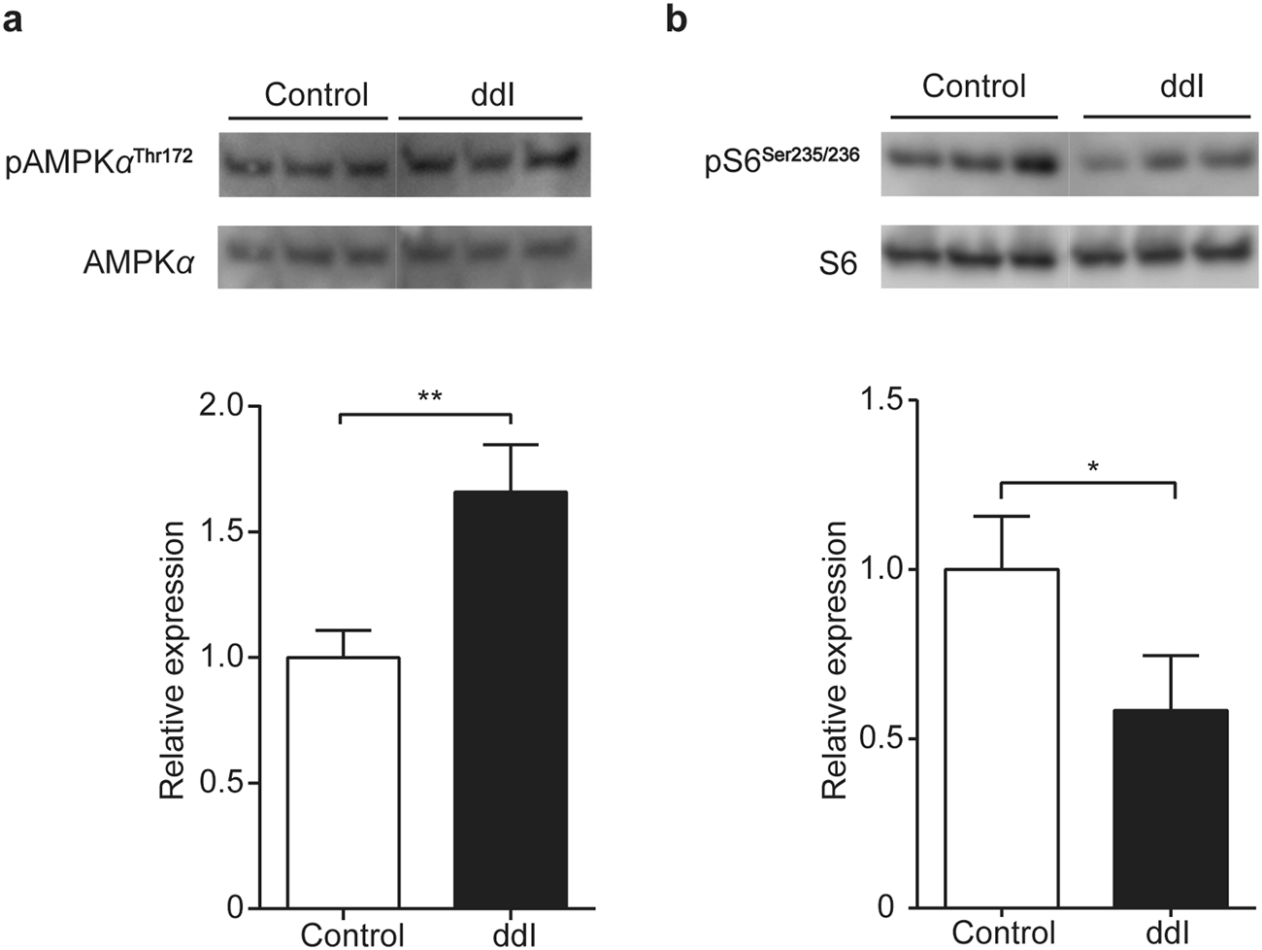
Protein expression from ddI-treated differentiated ARPE-19. Immunoblots (top) of protein lysates probed for AMPK (**a**) and mTOR (**b**) activation and quantification by densitometry (bottom) (p = 0.0062 in (**a**) and p = 0.0333 in (**b**)). Control (0 μM) or ddI (200 μM) treatment for 24 days. n = 3 for each group.

AMP-activated protein kinase (AMPK) is activated in response to an increase in the cellular AMP/ATP ratio, which can be caused by reduced cellular ATP. AMPK is activated by phosphorylation of the alpha subunit (AMPKα) and, in turn, regulates a number of key metabolic enzymes through phosphorylation. The ratio of phosphorylated AMPKα to total AMPKα was significantly increased in ddI-treated differentiated ARPE-19 cells (p = 0.0062) (Fig. 4a, full-length blots in Supplementary Figure 1), indicating activation of AMPK. AMPK is a well-known inhibitor of mechanistic target of rapamycin (mTOR). The relative amount of phosphorylated ribosomal protein S6 (pS6), a common gauge of mTORC1 activity, is reduced in ddI-treated ARPE-19 cells (p = 0.0333) (Fig. 4b, full-length blots in Supplementary Figure 1), indicating inhibition of this branch of the mTOR pathway.

### ddI-treated ARPE-19 cells exhibit increased resistance to oxidative stress

**Figure 5:**
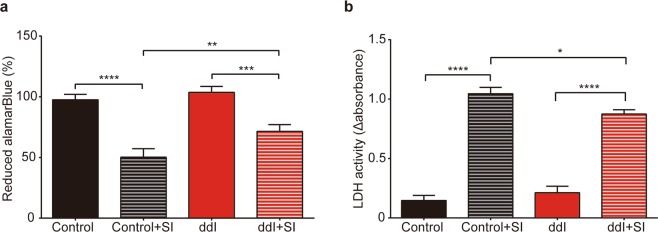
ddI-treated ARPE-19 and oxidative stress. Differentiated ARPE-19 cells were treated or not with 200 μM ddI for 24 days, and then challenged with 15 mM sodium iodate (SI) for 6 hours and assayed for cell viability (control vs. control + SI: p < 0.0001, ddI vs. ddI + SI: p = 0.0006, control + SI vs. ddI + SI: p = 0.0095) (**a**) and cytotoxicity (control vs. control + SI: p < 0.0001; ddI vs. ddI + SI: p < 0.0001; control + SI vs. ddI + SI: p = 0.0137) (**b**). n = 3 for each group; Two-way ANOVA followed by Bonferroni’s multiple comparisons was used.

Post-mitotic RPE cells are subject to a lifetime of high oxygen tension *in vivo*. Breakdown of the ability of RPE cells to resist oxidative stress has been implicated in the pathogenesis of AMD^42^. Sodium iodate is frequently used to model oxidative stress of the RPE *in vivo*^43–45^. We assessed the effect of ddI treatment on the ability of differentiated ARPE-19 cells to resist a strong, acute oxidative stress caused by sodium iodate. Sodium iodate decreased cell viability in control cells and in ddI treated cells. However, the sodium iodate insult had less of an effect in cells pretreated with ddI and resulted in more viability (Fig. 5a). Further, ddI pretreatment limited cell cytotoxicity (Fig. 5b).

## Discussion

Numerous studies have associated ddI with retinopathy in AIDS patients^24–28^, or with mitochondrial DNA damage in animal models^19,46,47^, but few studies have investigated the effects on differentiated cells in culture^22^, and none to our knowledge has described the use of NRTIs to induce mitochondrial dysfunction in RPE cells. Our approach provides a straightforward, specific, inexpensive, and non-toxic method to effect progressive loss of mitochondrial DNA in the RPE. In contrast to prior work, our approach is applicable to non-proliferating, differentiated RPE cells derived from a variety of sources^36,48,49^, and results in partial loss of mitochondrial DNA, which is likely a more informative model of RPE dysfunction in aging and disease.

A number of studies have implicated mutations of RPE mitochondrial DNA in the pathogenesis of AMD^7–9,50^, but the metabolic consequences of these mutations are unclear. Our results demonstrate that depletion of mitochondrial DNA in differentiated RPE cells in culture causes a decrease in MMP and a shift toward increased aerobic glycolysis. Consistent with this, gradual loss of OXPHOS capability in the murine RPE *in vivo* beginning in the early postnatal period results in an aerobic glycolytic phenotype and subsequent photoreceptor degeneration^44^. Interestingly, ARPE-19 cybrids with the AMD-risk-associated mitochondrial J haplotype have increased lactate secretion compared to cybrids with the protective H haplotype^51^. Increased lactate secretion is a characteristic of enhanced aerobic glycolysis and is equivalent to the increased ECAR we observed in mitochondrial-DNA-depleted differentiated RPE. Human RPE cells from aged donors exhibit high levels of oxidative stress and both reduced OXPHOS capability and aerobic glycolysis, conditions that may be similar to our sodium iodate treated cells^52^. Together, these findings suggest a process in which initial RPE mitochondrial dysfunction causes a shift toward glycolysis, and further degradation of function leads to cells that are metabolically exhausted.

Our results show that after 24 days of treatment with ddI differentiated ARPE-19 cells appeared to be better able to tolerate oxidative stress. This was an unexpected result considering the profound sensitivity of ρ^0^ ARPE-19 cells to a strong oxidant^11^. However, as rapamycin-mediated inhibition of mTORC1 activates autophagy and protects undifferentiated ARPE-19 cells from a lethal oxidative challenge^53^, our finding of increased resistance of ddI-treated, differentiated ARPE-19 cells to a lethal oxidative insult is consistent with this result and with others^54^. Together, these results suggest that moderate depletion of mitochondrial DNA triggers AMPK activation and increased resistance to oxidative damage, while more extreme degradation of mitochondrial energy production causes sensitivity to oxidants. It is possible that the ddI treatment is similar to the early stage of retinal degeneration, and the cells undergo corresponding pathophysiological changes to adapt to the process, thus better tolerating oxidative stress; but as the lesion progresses, oxidative stress exceeds the tolerable level of the cells, causing serious damage.

Our study has some limitations that should be understood before these results are considered clinically. We did not supplement extra uridine in the medium during the ddI experiments. As uridine and other pyrimidine nucleotides are needed for RNA-synthesis, glycosylation and membrane lipid synthesis, availability of uridine might have rescued some or all of the functional behavior of the retinal cells. However, there is also a suggestion that uridine may attenuate the toxicity of NRTIs. A study on antiretroviral pyrimidine analogues in adipose cells showed that adverse effects induced by stavudine, zidovudine, and zalcitabine, including mitochondrial DNA depletion, were prevented by uridine supplementation^55^. However, ddI had no effects in the preadipocytes of that study. In this study, mitochondrial DNA was depleted to around 40% of normal level not completely eliminated; therefore, there was likely to be some uridine production so, we avoided uridine supplementation in the cell culture media. It is not clear whether other nucleoside analogues would have shown similar results to ddI in this study, so these other nucleoside analogues should also be investigated in the future. Our results showed nuclear encoded NDUFA9 protein was reduced by 60%. This level of decrease suggests NDUFA9 nuclear encoded protein is not stable unless trafficked to the mitochondria and complexed with other respiratory proteins. Because NDUFA9 is involved in assembly of mitochondrial respiratory chain complex I and this complex involves 45 subunits, which are encoded by both nuclear and mitochondrial DNA. However, another possibility is a severe deficit in uridine occurred that caused a general reduction in all actively transcribed genes. Therefore, further experiments are needed to ensure that this is not due to a general deficiency of pyrimidines needed for RNA synthesis. Further studies, such as animal based experiments, are also needed to fully establish the clinical relevance of this study.

Our findings shed light on the mechanism of ddI-induced retinopathy and suggest that progressive loss of RPE mitochondrial function *in vivo* leads to increased glycolysis, and resistance to oxidative stress. Further exploration of the consequences of ddI-induced mitochondrial DNA depletion in differentiated cells could be a fruitful approach to understanding RPE pathogenesis in aging and disease.

## Supplementary information

Full-length blots of Figure 1c, Figure 4a, and Figure 4b

## Data Availability

The data set supporting the results of this article are included within the article.
