# Supplementary material for: Depletion of Mitochondrial DNA in Differentiated Retinal Pigment Epithelial Cells
Source: Sci Rep. 2019 Oct 25;9:15355. doi: 10.1038/s41598-019-51761-1 (PMC6814719; doi:10.1038/s41598-019-51761-1)

Depletion of Mitochondrial DNA in Differentiated Retinal Pigment Epithelial Cells

Xinqian Hu[1,2 *], Melissa A. Calton[2], Shibo Tang[3], Douglas Vollrath[2]

[1]State Key Laboratory of Ophthalmology, Zhongshan Ophthalmic Center, Sun Yat-sen University, Guangzhou, 510060, China
[2]Department of Genetics, Stanford University School of Medicine, Stanford, CA 94305, USA
[3]Aier Eye Institute, Aier School of Ophthalmology, Central South University, Changsha, Hunan, 410015, China.
*** Corresponding author**：xinqianhu@hotmail.com

Supplementary Figure 1: Full-length blots of Figure 1d, Figure 4a, and Figure 4b.

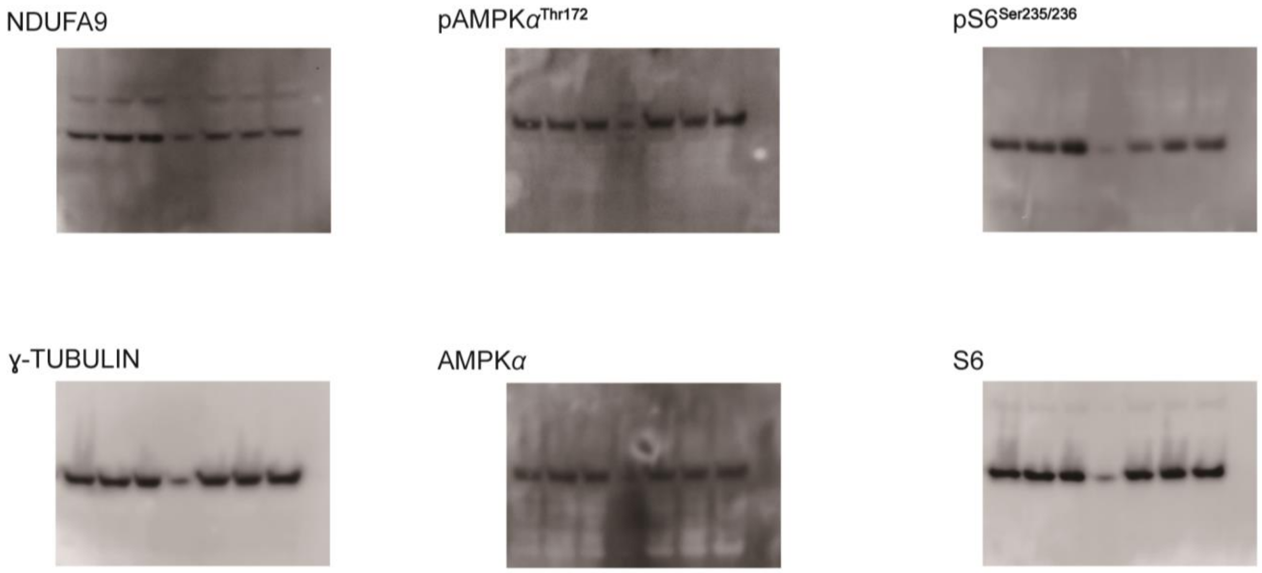

Supplement: Supplementary file 1 — Full-length blots of Figure 1c, Figure 4a, and Figure 4b [file 41598_2019_51761_MOESM1_ESM.pdf]
